# S-1 combined with docetaxel following doxorubicin plus cyclophosphamide as neoadjuvant therapy in breast cancer: phase II trial

**DOI:** 10.1186/1471-2407-13-583

**Published:** 2013-12-06

**Authors:** Yong Wha Moon, Soohyeon Lee, Byeong-Woo Park, Eun-Kyung Kim, Seung Il Kim, Ja Seung Koo, Seho Park, Min Jung Kim, Hyun Cheol Chung, Joo-Hang Kim, Joohyuk Sohn

**Affiliations:** 1 Yonsei Cancer Center, Divison of Medical Oncology, Department of Internal Medicine, Yonsei University College of Medicine, 50 Yonsei-ro, Seodaemun-gu, Seoul 120-752, Korea; 2 Department of General Surgery, Yonsei University College of Medicine, Seoul, Korea; 3 Department of Radiology, Yonsei University College of Medicine, Seoul, Korea; 4 Department of Pathology, Yonsei University College of Medicine, Seoul, Korea; 5 Current address: The University of Texas MD Anderson Cancer Center, Houston, TX, USA

**Keywords:** Breast cancer, Neoadjuvant chemotherapy, S-1, Docetaxel, Pathological complete response

## Abstract

**Background:**

This study evaluated the efficacy and safety of S-1 combined with docetaxel (SD) following doxorubicin plus cyclophosphamide (AC) as neoadjuvant therapy in patients with HER2-negative, stage II-III breast cancer.

**Methods:**

Patients received AC every 3 weeks for four cycles followed by S-1 (30 mg/m^2^ orally b.i.d. on days 1–14) and docetaxel (75 mg/m^2^ i.v. on day 1) every 3 weeks for four cycles. The primary endpoint was the pathological complete response (pCR) rate in breast and axillary lymph nodes.

**Results:**

The study included 49 patients with a median age of 43 years. The median breast tumor size was 4.0 cm by palpation. All patients were positive for involvement of axillary lymph node and five patients also had supraclavicular lymph node metastasis, which was confirmed by histological examination. In total, 85.4% of patients (41/49) completed eight cycles of therapy and 95.9% of patients (47/49) received curative surgery. The pCR rate was 22.5% (*n* = 11). The clinical response rate was 67.4%. During SD chemotherapy, the most frequent grade 3–4 toxicity was neutropenia (8.5% by cycle). There was a single treatment-related mortality from severe neutropenia. Grade 3 S-1 specific toxicities such as epigastric pain (12.2% by person), stomatitis (4.1% by person), and diarrhea (2.0% by person) were also observed. In particular, gastrointestinal discomfort led to dose reduction of S-1 in 45.8% of patients.

**Conclusions:**

Given all axillary lymph node positive diseases, neoadjuvant S-1 combined with docetaxel following AC showed a favorable anti-tumor activity but gastrointestinal discomfort should be carefully considered for future studies.

**Trial registration:**

NCT00994968

## Background

Neoadjuvant (pre-operative) chemotherapy is the standard of care in patients with inoperable locally advanced breast cancer, and is increasingly being considered for patients with operable disease [1]. Between pre- and postoperative chemotherapy, comparable clinical outcomes have been found, but in most studies breast-conserving surgery (BCS) was possible more often after preoperative treatment [2]. Combined doxorubicin and cyclophosphamide (AC) and sequential docetaxel is a widely accepted regimen for neoadjuvant chemotherapy based on the National Surgical Adjuvant Breast and Bowel Project Protocols (NSABPs) B-27 study, which demonstrated that sequential addition of docetaxel increased the rate of pathological complete response (pCR) compared with AC alone [3]. The pCR is known to be a short-term surrogate marker of neoadjuvant chemotherapy that strongly correlates with long-term clinical outcome [4]. Accordingly, one of the strategies to improve pCR is to add new compounds to established treatments.

S-1 is an orally bioavailable fluoropyrimidine antagonist composed of tegafur, a prodrug of 5-flurouracil (5-FU), combined with two modulators of 5-FU activity, 5-chloro-2,4-dihydroxypyridine (CDHP) and potassium oxonate. CDHP potentiates the effect of 5-FU by blocking the rapid catabolism of 5-FU into inactive metabolite. Potassium oxonate preferentially localizes in the gut where it inhibits local activation of 5-FU, thereby decreasing 5-FU-related gastrointestinal toxicity [5]. Clinically, S-1 has been widely approved for gastric cancer treatment in many countries in Asia and Europe [6] and for several other cancers in Japan [7]. In a phase 2 clinical trial (*n* = 111), S-1 monotherapy showed activity and tolerability in metastatic breast cancer with a response rate of 42%, warranting further research on the role of S-1 in breast cancer [8].

A S-1 plus docetaxel combination was reported to have a synergistic antitumor effect in a breast cancer xenograft study, suggesting partly through significant down-regulation of the activity of dihydropyrimidine dehydrogenase, the rate-limiting enzyme in the catabolism of 5-FU [9]. However, the mechanism underlying the synergism of these compounds is not fully understood. Although a S-1 and docetaxel combination was active and tolerable in several phase II trials of gastric cancer [10] and non-small cell lung cancer patients [11], a clinical study of this combination therapy in breast cancer patients has not been published to date.

Based on these results, the present phase II trial was designed to evaluate the efficacy and the safety of a combination of S-1 and docetaxel following AC chemotherapy as neoadjuvant treatment in stage II-III breast cancer.

## Methods

### Patient eligibility

Women with previously untreated clinical stage II or III breast cancer were eligible for this neoadjuvant trial if the following eligibility criteria were met: i) pathologically confirmed invasive ductal or lobular carcinoma from a core biopsy specimen; ii) HER2-negativity of 0 or 1+ by immunohistochemistry, or HER2 non-amplification by fluorescent in situ hybridization; iii) age ≥18 years; iv) Eastern Oncology Cooperative Group performance status of ≤1; v) adequate cardiac function (left ventricular ejection fraction > 50%); vi) adequate bone marrow (neutrophils ≥1.5 × 10^3^/μl, platelets ≥100 × 10^3^/μl, Hb ≥10.0 g/dl), renal function (serum creatinine ≤1.5 times the upper normal limit or creatinine clearance ≥ 50 ml/min by Cockroft formula), and liver function (serum bilirubin ≤1.5 times the upper normal limit, aspartate aminotransferase/alanine aminotransferase ≤2.5 times the upper normal limit) within 2 weeks before starting the therapy; and vii) no previous chemo-, radio- or hormone therapy. Patients were excluded if they met the following criteria: i) T4d/inflammatory breast cancer; ii) potentially or currently pregnant or lactating; iii) taking medications that alter the pharmacokinetics of S-1 (for example, allopurinol, phenytoin); or iv) inability to swallow S-1 tablets or malabsorptive gastrointestinal condition.

All patients provided written informed consent and this study was approved by the Institutional Review Board of Severance Hospital, Seoul, Korea.

### Study treatment

This was a single-arm, single-center, phase II study of S-1 combined with docetaxel (SD) following doxorubicin plus cyclophosphamide (AC) as neoadjuvant chemotherapy in patients with stage II-III breast cancer. Neoadjuvant chemotherapy schedule was as follows: For the initial AC treatment, doxorubicin (60 mg/m^2^ i.v. on day 1) and cyclophosphamide (600 mg/m^2^ i.v. on day 1) were administered every 3 weeks for four cycles. Upon completion of AC treatment, if the diseases had not progressed by physical and radiological examinations and toxicities were acceptable, SD treatment with S-1 (30 mg/m^2^ orally b.i.d. on days 1–14) and docetaxel (75 mg/m^2^ i.v. day 1) was given every 3 weeks for four cycles.

Modifications of doses and dosing schedules were as follows: If the neutrophil count was ≥1.5 × 10^3^/μl and the platelet count was ≥100 × 10^3^/μl, we would begin the next cycle for both AC and SD. If these values were not reached, AC or SD was delayed by 1 week. At the end of the first week of delay, if the neutrophil count was 1.0–1.5 × 10^3^/μl and the platelet count was 75–100 × 10^3^/μl, the next doses of doxorubicin/cyclophosphamide and S-1/docetaxel were reduced by 1 step to 50/500 mg/m^2^ and 25/60 mg/m^2^, respectively. If the neutrophil count was ≤1.0 × 10^3^/μl or the platelet count was ≤75 × 10^3^/μl, therapy was delayed for one more week. After the second week of delay, if the neutrophil count was ≥1.5 × 10^3^/μl and the platelet count was ≥100 × 10^3^/μl, AC and SD were reduced by 1 step; however, if these values were not reached, the trial would be stopped. Granulocyte colony-stimulating factor (G-CSF) was administered in the case of febrile neutropenia. Prophylactic G-CSF usage was permitted from the second cycle. Regarding non-hematological toxicity, if grade 3–4 S-1 specific toxicities such as diarrhea, stomatitis, or epigastric pain were observed, S-1 was omitted and restarted at a 1-step lower dose when toxicities were recovered to grade 1 or baseline; however, docetaxel administration was continued. If grade 3–4 peripheral neuropathy was observed, docetaxel was omitted and restarted at a 1-step lower dose when toxicities recovered to grade 1 or baseline. If grade 3–4 abnormalities of serum bilirubin, alkaline phosphatase, aspartate aminotransferase, or alanine aminotransferase increase were observed, docetaxel and S-1 were delayed and restarted at a 1-step lower dose when toxicities recovered to grade 1 or baseline. If the other grade 3–4 non-hematological toxicities were observed, AC or SD could be delayed and reduced according to the investigator’s discretion.

### Post-study treatment

The type of breast surgery after neoadjuvant chemotherapy was determined by using a multidisciplinary team approach considering the clinical and radiological response to neoadjuvant chemotherapy, presence of multifocal or multicentric tumor, and patient preference. All patients with initial axillary node-positive disease underwent standard level I/II axillary lymph node dissection with or without explorative sentinel lymph node biopsy. Patients with Initially cytology-proven supraclavicular lymph node metastasis underwent supraclavicular lymph node dissection. After surgery, all patients received adjuvant radiotherapy covering the whole breast and regional nodes including supraclavicular area. Patients with hormone receptor-positive tumors received adjuvant endocrine therapy. However, postoperative adjuvant chemotherapy was not scheduled. If tumors were not resectable, patients were taken off the clinical trial.

### Study endpoints

The primary endpoint was the pCR rate, defined as the absence of invasive carcinoma in the breast and no involvement of carcinoma to axillary nodes (AXLNs) (i.e., pathological stage T0N0 or TisN0). Secondary endpoints were the clinical response rate, safety, pCR rate in the breast regardless of AXLN, and BCS rate.

### Assessments of endpoints

Tumor measurement by mammography, ultrasonography, and/or MRI was scheduled at baseline, at the completion of four cycles of AC, and before surgery. The breast and regional lymph nodes were examined by palpation every cycle. A PET-CT scan was performed at baseline and at the completion of chemotherapy to exclude distant metastases. Pathological responses of the breast tumor and infiltration of regional lymph nodes were assessed and staged according to the TNM system. The clinical response was defined according to the Response Evaluation Criteria for Solid Tumors (version 1.1) [12]. Toxicity was graded based on the National Cancer Institute’s Common Terminology Criteria for Adverse Events version 3.0. For pathological assessment, excised tissues from the breast were cut to 5-mm thickness and those from the regional lymph nodes to 2-mm thickness. Patients were considered to have had BCS if the final surgical procedure was tumorectomy, segmentectomy, or quadrantectomy.

### Statistical analysis

The null hypothesis is that the pCR rate is 11%. This pCR rate was estimated lower than 14.3% obtained by docetaxel following by AC in GEPARDUO study in which only operable T2-3 and N0-2 tumors were enrolled but our study enrolled more advanced stages with pathologically confirmed axillary node involvement (T1-4c and N1-3) including inoperable, stage III tumors [13]. The alternative hypothesis is that addition of S-1 in combination with docetaxel will increase the pCR rate to 25%. With a two-sided significance level of 0.05 and a power of 0.8 to reject the null hypothesis, the minimum sample size was determined to be 44. Assuming a 10% of drop-out rate, the final sample size was calculated to be 49 patients. An optimal cut-off point for Ki67 was determined as 30% using the minimum P value approach in the analysis of predictors of pCR.

## Results

### Patient characteristics

From July 2009 to September 2011, 49 patients were enrolled in this study at Severance Hospital, Yonsei University Health System, Seoul, Korea. All 49 patients received at least one dose of study treatment and were included in the evaluation of pCR and toxicity [intention to treat (ITT) population]. Baseline characteristics of the 49 patients are summarized in Table 1. All patients were positive for involvement of axillary lymph node and five patients also had supraclavicular lymph node (SCL) metastasis. Involvement of axillary and supraclavicular node was confirmed by fine needle aspiration and histological examination.

**Table 1:** Patient characteristics (ITT population)

**Characteristics**	***n***	**(%)**
Number of total patients	49	
Median age, years (range)	43 (27–60)	
Menopausal status		
Premenopause	42	(85.7%)
Postmenopause	7	(14.3%)
Histology		
Invasive ductal carcinoma	46	(93.9%)
Invasive lobular carcinoma	1	(2.0%)
Mixed type (ductal + lobular)	2	(4.1%)
ECOG performance status		
0	25	(51.0%)
1	24	(49.0%)
Prechemotherapy T stage		
cT1	5	(10.3%)
cT2	37	(75.5%)
cT3	6	(12.2%)
cT4	1	(2.0%)
Prechemotherapy N stage		
cN0	0	(0.0%)
cN1	30	(61.2%)
cN2	14	(28.6%)
cN3	5	(10.2%)
AJCC clinical stage		
IIA	3	(6.1%)
IIB	25	(51.0%)
IIIA	16	(32.7%)
IIIB	0	(0.0%)
IIIC	5	(10.2%)
Median tumor size by palpation, cm (range)	4.0 (0–11.0)	
Hormonal receptor status^*^		
Positive for ER or PgR	36	(73.5%)
Negative for ER and PgR	13	(26.5%)
HER2 status		
Positive	0	(0.0%)
Negative	49	(100%)

*ECOG*, Eastern Cooperative Oncology Group; *ER*, estrogen receptor; *PgR*, progesterone receptor. ^*^The cut-off of ER and PgR positivity was 10% stained cells by immunohistochemistry.

### Overview of treatment results

Forty-one patients (83.7%) completed four cycles of AC and four cycles of SD chemotherapy and also underwent surgery. Among eight patients (16.3%) who could not complete the scheduled chemotherapy, one was lost to follow-up during AC cycles and seven were taken off the study during SD cycles for the following reasons: two withdrew consent after 1 cycle; three exhibited toxicity after 1, 2, or 3 cycles; one had disease progression after 2 cycles; one died from septic shock after 2 cycles. Six of the seven patients who dropped out during SD cycles received surgery.

### Efficacy outcomes

Efficacy outcomes are summarized in Table 2. The primary endpoint, the rate of pCR in both breast and AXLN, was 22.5% (*n* = 11) in the ITT population. The rate of pCR in the breast and in AXLN was 30.6% (*n* = 15) and 40.8% (*n* = 20), respectively. In the per protocol population, which excluded patients who were lost to follow-up (*n* = 1) or withdrew consent (*n* = 2), the rate of pCR in the breast and AXLN was 23.9% (11/46). The rate of BCS was 42.9% (21/49). The pCR rate in the breast and AXLN was 30.8% (4/13) for the triple-negative disease and 19.4% (7/36) for hormone receptor-positive disease.

**Table 2:** Summary of efficacy outcomes

	**ITT population ( *n *** **= 49)**	
**Endpoints**	***n***	**(%)**
pCR^*^ in breast and AXLN	11	(22.5%)
pCR^*^ in breast	15	(30.6%)
Clinical response		
Complete response	6	(12.3)
Partial response	27	(55.1)
Stable disease	14	(28.6)
Progressive disease	1	(2.0)
Non-assessable	1	(2.0)
Breast-conserving surgery		
Yes	21	(42.9)
No	26	(53.1)
Non-assessable	2	(4.0)

*ITT*, intent-to-treat; *pCR*, pathological complete response; ^*^pCR was defined as the absence of invasive disease.

Clinical response was observed in 67.4% of patients (6 complete and 27 partial responders among 49 patients). Fourteen patients (28.6%) showed stable disease and one patient (2.0%) showed progressive disease. According to the site of the tumor, the clinical response rates were 67.4% in the breast (7 complete and 26 partial responders), 55.1% in the AXLN (27 complete responders), and 40.0% in the SCL (2 complete responders out of 5 with SCL metastasis). Subsequent clinical response to SD chemotherapy was evaluated in 31 patients who had serial sonographs. The median tumor size in the breast was 26 mm, 13 mm, and 10 mm at baseline, completion of AC, and completion of SD chemotherapy, respectively. Compared to tumor size before SD chemotherapy, subsequent clinical response rates in the breast to SD chemotherapy were: complete response, 6.9% (2/29); partial response, 31.0% (9/29); stable disease, 62.1% (18/29). Two complete responses to AC chemotherapy were maintained after subsequent SD chemotherapy.

In an exploratory finding, tumors with high Ki-67 (≥30%) or small initial size of tumor (≤2 cm) showed a trend toward a higher pCR rate than those without but they did not reach statistical significance (Table 3).

**Table 3:** Univariate analysis for predictors of pathologic complete response

**Variables**		***N***	**pCR *n *** **(%)**	**Non-pCR *n *** **(%)**	***P*** value^*^
Ki-67	<30%	15	2 (13.3)	13 (86.7)	0.070
	≥30%	18	8 (44.4)	10 (55.6)	
Initial tumor size	≤2 cm	7	4 (57.1)	3 (42.9)	0.083
	>2 cm	32	7 (21.9)	25 (78.1)	
Initial clinical stage	II	22	5 (22.7)	17 (77.3)	0.482
	III	17	6 (35.3)	11 (64.7)	
Age, year	< median (43)	20	7 (35.0)	13 (65.0)	0.480
	≥ median (43)	19	4 (21.1)	15 (78.9)	
HR (10% of cut-off)	Negative	11	4 (36.4)	7 (63.6)	0.694
	Positive	28	7 (25.0)	21 (75.0)	

*HR*, hormone receptor. ^*^Chi-square or Fisher’s exact test.

### Safety

All grades of hematologic (by cycle) and non-hematologic (by patient) toxicities are listed in Table 4. Toxicities of AC chemotherapy were mainly grade 1–2 nausea, vomiting and anorexia as expected. In the SD cycles, the dominating grade 3–4 toxicity was myelosuppression and epigastric pain. Grade 3–4 hematological toxicities were neutropenia (8.5%) and febrile neutropenia (2.9%). After two cycles of SD, one neutropenic patient experienced infection that led to death. S-1 specific toxicities were as follows: epigastric pain, 20.4% at grade 1–2 and 12.2% at grade 3; stomatitis, 28.6% at grade 1–2 and 4.1% at grade 3; diarrhea, 8.2% at grade 1–2 and 2.0% at grade 3; hand-foot syndrome, 8.2% at grade 1–2 and 2.0% at grade 3.

**Table 4:** Toxicity profiles of AC/SD chemotherapy

		**NCI-CTC AE grade**					
**Toxicities**		**G1**	**G2**	**G3**	**G4**	**G5**	**≥G3 (%)**
Hematologic toxicity (by cycle)							
	Neutropenia	2/1	1/3	6/6	6/9	0/0	12/15 (6.2/8.7)
	Febrile neutropenia	0/0	0/0	6/4	0/1	0/0	6/5 (3.1/2.9)
	Anemia	0/0	0/0	0/1	2/0	0/0	2/1 (1.0/0.6)
Non-hematologic toxicity (by patient)							
	Epigastric pain	7/15	3/2	0/6	0/0	0/0	0/6 (0/12.2)
	Nausea	19/11	8/4	1/2	0/0	0/0	1/2(2.0/4.1)
	Vomiting	8/9	4/3	1/2	0/0	0/0	1/2 (2.0/4.1)
	Stomatitis/mucositis	12/14	2/10	0/2	0/0	0/0	0/2 (0/4.1)
	Infection	0/0	1/1	1/0	0/1	0/1	1/2(2.0/4.1)
	Increased ALT	0/0	0/2	1/2	0/0	0/0	1/2 (2.0/4.1)
	Myalgia	6/13	1/7	0/1	0/0	0/0	0/1 (0/2.0)
	Diarrhea	2/4	2/2	0/1	0/0	0/0	0/1 (0/2.0)
	Hand-foot syndrome	0/1	0/3	0/1	0/0	0/0	0/1 (0/2.0)
	Anorexia	18/10	3/9	0/0	0/0	0/0	0/0 (0/0)
	Constipation	9/8	1/7	0/0	0/0	0/0	0/0 (0/0)
	Arthralgia	1/2	1/3	0/0	0/0	0/0	0/0 (0/0)
	Peripheral neuropathy	2/6	0/3	0/0	0/0	0/0	0/0 (0/0)
	Edema	1/9	0/1	0/0	0/0	0/0	0/0 (0/0)
	Fatigue	16/0	0/1	0/0	0/0	0/0	0/0 (0/0)

*NCI-CTC AE*, National Cancer Institute-Common Terminology Criteria for Adverse Events. Total number of cycles delivered was 193/172 cycles for AC/SD.

### Treatment delivery and dose intensity

Since AC chemotherapy is a routine treatment and SD chemotherapy is experimental, dose-intensity analysis focused on SD chemotherapy. In total, 172 cycles of SD chemotherapy were delivered. Docetaxel was delayed in 7 cycles (4.1%), mainly due to neutropenia. At least one dose of S-1 was omitted in 27 cycles (15.7%) due to stomatitis (6 cycles), epigastric pain (4 cycles), and other toxicities (17 cycles). Of 48 patients who received at least one cycle of SD chemotherapy, 12 patients (25%) required dose reduction of both docetaxel and S-1 due to infection (*n* = 2), neutropenia (*n* = 2), asthenia (*n* = 2), and other toxicities (*n* = 6). Two patients (4.2%) required dose reduction in only docetaxel and 10 patients (20.8%) in only S-1, mainly due to epigastric pain (*n* = 7). Five of these 10 patients discontinued S-1 completely after 1 (*n* = 1), 2 (*n* = 2), or 3 cycles (*n* = 2). The mean relative dose intensity (RDI) of docetaxel and S-1 was 91.4% and 79.6%, respectively.

## Discussion

The current study evaluated the efficacy and tolerability of S-1 in combination with docetaxel following AC chemotherapy as neoadjuvant treatment in patients with HER2-negative, stage II-III breast cancer. To the best of our knowledge, this is the first neoadjuvant trial to test a docetaxel plus S-1 combination in breast cancer patients. Given all patients had pathologically confirmed axillary lymph node involvements, this novel combination showed activity with a favorable pCR rate (22.5%) in both breast and axillary nodes, although the primary endpoint, pCR, did not reach the predetermined threshold level (25%). Further, gastrointestinal discomfort should be carefully considered for future studies. However, concomitant addition of S-1 did not decrease the dose intensity of docetaxel.

Comparing cross-study efficacy of chemotherapy needs special attention regarding the design of the studies such as patient population, definition of endpoint, and dose intensity. As shown in Table 5, in the NSABP B-27 study, AC-D produced a similar pCR rate in the breast and lymph nodes (21.8%) to our study (22.5%); however, clinical nodal involvement (30%) was less frequent than in our study (100%). In addition, NSABP B-27 did not enroll patients with cN2-3, whereas such patients represented 38.8% of our study population. The tumor size was similar between the two studies. In the GEPARDUO study [13], which is another phase III neoadjuvant study using AC-D regimen, the pCR rate in the breast with AC-D (22.3%) was lower than the pCR rate in our data (30.6%) and both studies had comparable median tumor size (4 cm). In terms of dose intensity, docetaxel delivery was scheduled at a higher dose in the prior two studies (100 mg/m^2^) than in our study (75 mg/m^2^).

**Table 5:** Comparison of neoadjuvant trials with AC followed by docetaxel with or without S-1

**Trials**	**Patients**			**Regimen**	**pCR**			
		**Tumor**	**cLN+**		**Breast + LN**		**Breast**	
	**Stage**	**>4 cm**	**(%)**		+CIS^*^	–CIS^**^	**+CIS**	**–CIS**
This study	T1-4c, N1-3	45%	100%	AC→SD	22.5%	NA	30.6%	22.5%
NSAPB B-27 [3]	T1c-3, N0-1	45%	30%	AC	11.5%	NA	13.7%	9.6%
		45%	30%	AC→D	21.8%	NA	26.1%	18.9%
GEPARDUO [13]	T2-3, N0-2	32%	38%	AD	NA	7.0%	11.0%	7.0%
		33%	42%	AC→D	NA	14.3%	22.3%	15.9%

*A*, doxorubicin; *C*, cyclophosphamide; *CIS*, carcinoma in situ; *cLN*, clinically detected lymph node; *D*, docetaxel; *NA*, non-assessable; *S*, S-1. ‘^*^ + CIS’ or ‘^**^–CIS’ indicates that the definition of pCR includes or does not include CIS.

Based on reports that capecitabine, which is another prodrug of 5-FU, in combination with docetaxel showed superior efficacy over docetaxel monotherapy in patients with metastatic breast cancer [14], adjuvant and neoadjuvant trials were conducted with a combination of capecitabine and docetaxel. However, combination treatment did not result in significantly improved pCR rates in the neoadjuvant GEPARQUATTRO [15] and NSABP B-40 trials [16]. In the adjuvant FinXX trial [17], integration of capecitabine into an adjuvant regimen that contained docetaxel, epirubicin, and cyclophosphamide did not significantly improve recurrence-free survival compared with a similar regimen without capecitabine. Exploratory subset analysis of the neoadjuvant GEPARQUATTRO study showed that in cT4 tumors (*n* = 152) a capecitabine plus docetaxel combination produced a higher pCR rate (18.9%) than doctaxel monotherapy (5.1%). In subset analysis of the adjuvant FinXX trial, in patients with triple-negative disease or more than three metastatic axillary lymph nodes the addition of capecitabine improved recurrence-free survival. Taken together, these results suggest that the efficacy benefit of capecitabine may be limited to patients with a high risk of breast cancer recurrence when used in neoadjuvant and adjuvant settings.

In the current study, SD following AC produced a higher pCR rate in triple-negative disease than in hormone receptor-positive disease. Considering triple-negative disease, which is biologically more aggressive, is generally more chemosensitive to neoadjuvant chemotherapy [18], we cannot exclude the possibility that our regimen might be more beneficial in triple-negative disease. Further studies to incorporate S-1 into docetaxel following AC might be worthwhile in patients with a relatively high risk of recurrence including triple-negative disease.

In determining doses of S-1 and docetaxel we did not want to compromise the dose of docetaxel, which is an approved active drug in the neoadjuvant setting of breast cancer. We therefore fixed the dose of docetaxel at 75 mg/m^2^ in 3 weeks, which is an effective and tolerable dose in Korean patients [19]. As recommended in a phase I trial of S-1 and docetaxel conducted in gastric cancer [20], we determined the dose of S-1 to be 30 mg/m^2^ b.i.d. instead of 35 mg/m^2^ b.i.d. for 14 days every 3 weeks, which is a widely accepted dose for monotherapy in Korea [21]. At these doses and dosing schedules, grade 3–4 neutropenia was not frequent (8.5% by cycle) and was mostly manageable except for a single case of treatment-related death from severe neutropenia. However, gastrointestinal discomfort caused by S-1 was a hurdle to administer S-1 in some patients. It is noteworthy that 20.8% of patients (*n* = 10) needed dose reduction of S-1, and five of these (10.4% of total patients) stopped S-1 treatment due to intolerability. Despite this, the RDI of docetaxel (91%) could be maintained at a high level by omitting or reducing only S-1 when S-1 specific toxicities were encountered. The RDI of S-1 was down to 80% with the strict modification in dosing of S-1,

## Conclusion

In conclusion, given all axillary lymph node positive diseases, S-1 combined with docetaxel following AC showed a favorable anti-tumor activity, although the primary endpoint was not met. Further, gastrointestinal discomfort should be carefully considered for future studies.

## Competing interests

The authors declare that they have no competing interests. This research was funded by Sanofi-Aventis.

## Author’s contributions

YWM designed the study, analyzed the data, and drafted the manuscript. SL collected and analyzed the data. BWP, SIK and SP were involved in the study design and enrolled patients. EKK and MJK were involved in the study design and carried out radiological response assessment. JSK was involved in the study design and carried out pathological response assessment. HCC and JHK coordinated the study. JHS conceived of the study, enrolled patients, helped to draft the manuscript, and coordinated the study. All authors read and approved the final manuscript.

## Pre-publication history

The pre-publication history for this paper can be accessed here:

http://www.biomedcentral.com/1471-2407/13/583/prepub
